# The Reformed Public House

**Published:** 1924-07

**Authors:** D. C. Dering


### CORRESPONDENCE.

### What is the Voluntary Principle?

*To the Editor of* The Hospital and Health Review.

### The Reformed Public House.

*To the Editor of* The Hospital and Health Review.

Sir,—Mr. Simons, who writes on behalf of the Society known as the True Temperance Association, suggests that the Public House Improvement Bill was an attempt to induce publicans to keep to their part of the bargain—in other words, to fulfil their duty to the public. It is probably more true to say that the Bill was designed to increase the facilities for the sale of beer and other alcoholic drinks. Otherwise, the inducements to qualify for special remissions are not very substantial, seeing that the structural alterations would in many cases involve very considerable outlay on the part of the brewery owning the licensed house. If the Bill had proved to be a *bonâ fide* measure, and not open to the criticisms given above, it would be another matter.

The Carlisle scheme has reduced the licensed houses in that city by nearly fifty per cent., to say nothing of other valuable action, and I should say that the facilities have certainly not been increased. The Carlisle scheme and the Bill which Mr. Simons advocates are two very different matters. The former is based on the elimination of private profit, while the latter is designed to strengthen the position of the liquor trade in the country, a proceeding which I cannot support.

D. C. Dering.

[This correspondence is now closed.—*Editor*, The Hospital and Health Review.]

